# Macular Choriocapillaris and Retinal Microvascular Alterations Across the Prediabetes–Early Type 2 Diabetes Continuum

**DOI:** 10.1167/iovs.67.10.16

**Published:** 2026-08-06

**Authors:** Fang Zheng, Yueheng Hong, Jacqueline Chua, Shu Hui Foo, Kanae Fukutsu, Yong Mong Bee, Leopold Schmetterer, Bingyao Tan, Gavin S. W. Tan

**Affiliations:** 1 Eye Center, The Second Affiliated Hospital Zhejiang University School of Medicine, Hangzhou, China; 2 Singapore Eye Research Institute and Singapore National Eye Centre, Singapore, Singapore; 3 Ophthalmology and Visual Sciences Academic Clinical Program, Duke-National University of Singapore (NUS) Medical School, Singapore, Singapore; 4 SERI-NTU Advanced Ocular Engineering (STANCE), Nanyang Technological University, Singapore, Singapore; 5 Department of Ophthalmology, Faculty of Medicine and Graduate School of Medicine, Hokkaido University, Sapporo, Japan; 6 Department of Endocrinology, Singapore General Hospital, Singapore, Singapore; 7 Medicine Academic Clinical Programme, Duke-National University of Singapore (NUS) Medical School, Singapore, Singapore

**Keywords:** macular microvascular, choriocapillaris, prediabetes, type 2 diabetes, swept-source optical coherence tomography angiography

## Abstract

**Purpose:**

To characterize macular microvascular alterations across the dysglycemia spectrum, from prediabetes (pre-DM) to early type 2 diabetes (T2DM), before clinically detectable diabetic retinopathy develops.

**Methods:**

In this cross-sectional study, participants with pre-DM, recent-onset T2DM, and age-matched healthy controls underwent swept-source optical coherence tomography angiography. Quantified metrics included vessel density (VD), perfusion density (PD), foveal avascular zone acircularity, and choriocapillaris flow-deficit (FD) density and size. One eye per participant was analyzed. Feature importance was assessed using a random forest model with SHAP values.

**Results:**

Analysis of 309 eyes (79 controls, 85 pre-DM, 75 T2DM <3 years, and 70 T2DM 3–7 years) showed that microvascular impairment is evident from the prediabetic stage. Compared with controls, participants with prediabetes had lower VD and PD in both superficial and deep capillary plexuses, together with higher choriocapillaris FD density (all *P* < 0.05). Retinal capillary metrics were similar between pre-DM and early T2DM (all *P* > 0.05), whereas choriocapillaris impairment progressed across dysglycemia stages, with large-size FD density increasing from pre-DM to T2DM <3 years and T2DM 3–7 years (9.3 ± 0.9 vs. 9.7 ± 1.1 and 9.8 ± 1.0, *P* < 0.05). Among OCTA metrics, choriocapillaris FD density showed the strongest discriminatory ability and was the most influential feature in SHAP analysis.

**Conclusions:**

Macular microvascular impairment begins in prediabetes. Retinal capillary changes plateau early, whereas choriocapillaris impairment progresses with worsening dysglycemia, suggesting greater sensitivity of choriocapillaris metrics for monitoring early metabolic disease.

The management of diabetes and its long-term multi-organ complications remains a substantial and growing global health challenge. Prediabetes, defined as impaired glucose tolerance (IGT) or impaired fasting glucose (IFG), represents a high-risk state for progression to type 2 diabetes. Globally, millions of adults are living with prediabetes, and without timely intervention, a large proportion will eventually develop diabetes.^1^^,^^2^ Evidences suggest that even in the prediabetic stage, prior to clinically detectable organ dysfunction, microvascular abnormalities are already present in peripheral microvascular beds, including those of the skin, skeletal muscle, kidney, and retina.^3^^–^^8^ Among these tissues, the retina provides a uniquely optically accessible window to observe microvascular alterations at the level of terminal capillary networks.

Optical coherence tomography angiography (OCTA) enables noninvasive, depth-resolved visualization of three-dimensional retinal microvascular structures, including the superficial capillary plexus (SCP) and deep capillary plexus (DCP).^9^ Swept-source OCTA (SS-OCTA), by using a longer wavelength, further enhances imaging penetration through the retinal pigment epithelium (RPE), allowing for improved visualization of the choriocapillaris, an area not well captured by spectral-domain OCTA (SD-OCTA).^10^ Beyond qualitative visualization, OCTA also provides quantitative microvascular metrics such as vessel density (VD), perfusion density (PD), and flow deficits (FD), enabling objective characterization of microvascular morphology.^11^^,^^12^

The progression from diabetes onset to clinically detectable diabetic retinopathy (DR) typically spans several years.^13^^–^^15^ However, microvascular injury begins much earlier and occurs silently before overt DR becomes apparent. Previous studies have shown that reductions in retinal capillary density can be detected prior to the clinical onset of DR.^16^^,^^17^ Within the first year after diabetes diagnosis, macular perfusion in the SCP can already be reduced, while prolonged disease duration contributes to impairment in both SCP and DCP in the retina.^18^ Moreover, multiple studies have reported reduced VD and PD of retinal capillaries even in individuals with prediabetes.^4^^–^^8^ However, findings related to foveal avascular zone (FAZ) morphology have been inconsistent across studies.^19^ Importantly, choriocapillaris alterations, representing a critical terminal capillary bed that may be particularly vulnerable to metabolic stress, remain insufficiently investigated in prediabetes and early diabetes.

In this study, we aimed to characterize both retinal and choriocapillaris alterations as glycemic status progresses from normoglycemia, through prediabetes, to early diabetes, using SS-OCTA for microvascular quantification, combined with machine-learning assisted statistical analysis. We also tested the hypothesis that choriocapillaris abnormalities may arise earlier and perhaps more predominantly than retinal microvascular impairment in the prediabetic and early diabetic stages.

## Methods

### Study Population

This cross-sectional observational study included participants from two independent cohorts: individuals with prediabetes and recent-onset diabetes enrolled in the Pre-DICTED EYE study (The Pre-Diabetes Interventions and Continued Tracking to Ease Out Diabetes trial), and age-matched healthy controls from the SIENA (Singapore Imaging Eye Network) study. Both studies were conducted at the Singapore National Eye Centre in accordance with the Declaration of Helsinki and were approved by the SingHealth Centralised Institutional Review Board (Pre-DICTED EYE: NO. 2023–2659; SIENA: No. 2018–2020). Demographic information, including age, sex, and ethnicity, was collected using standardized questionnaires. Blood pressure, body height, weight and axial length were measured during each visit. Axial length was measured using non-contact optical biometry (IOLMaster 700; Carl Zeiss Meditec, Jena, Germany).

The Pre-DICTED EYE study recruited participants from the Pre-DICTED clinical trial (NCT03503942), where individuals were followed for three years with semiannual fasting plasma glucose (FPG) testing and annual oral glucose tolerance testing (75*g* OGTT). Diabetes mellitus was diagnosed following WHO and ADA criteria (FPG ≥ 7.0 mmol/L or 2h-PG ≥ 11.1 mmol/L or Hemoglobin A1c ≥ 6.5%), and the date of conversion to diabetes was recorded. Details of study methodology have been previously published.^20^ In both Pre-DICTED EYE and SIENA studies, two 45° fundus photographs, one centered on the macula and one on the optic disc, were acquired for each eye using a digital retinal camera (Maestro, Centervue, Padova, Italy) after pharmacological dilation with 1% tropicamide. Images were graded by certified graders at the Singapore Ocular Reading Centre for the presence of DR or other retinal pathology.

### SS-OCTA Image Acquisition

All participants underwent SS-OCTA imaging using the PLEX Elite 9000 system (Carl Zeiss Meditec, Dublin, CA). The device operates at 100,000 A-scans/second with a 1,050-nm central wavelength, ∼5 µm axial resolution, and ∼14 µm lateral resolution. Motion artifacts were minimized using FastTrac tracking. Each eye was imaged using a 3 × 3 mm^2^ scan centered on the fovea, with 300 A-scans per B-scan, repeated four times across 300 B-scan positions, producing a uniform sampling grid of 10 µm spacing.

Automated software generated en-face images of the SCP and DCP. The choriocapillaris slab was defined as 31–40 µm below the RPE with manual adjustment.^21^ Projection artifacts from retinal vessels were automatically suppressed using the proprietary projection artifact removal algorithm implemented in the PLEX Elite 9000 review software. The resulting projection artifact resolved choriocapillaris en face images were used for subsequent flow deficit analysis, without any additional manual masking or exclusion of these regions.

### Quantification of SS-OCTA Images

**Figure 1.**
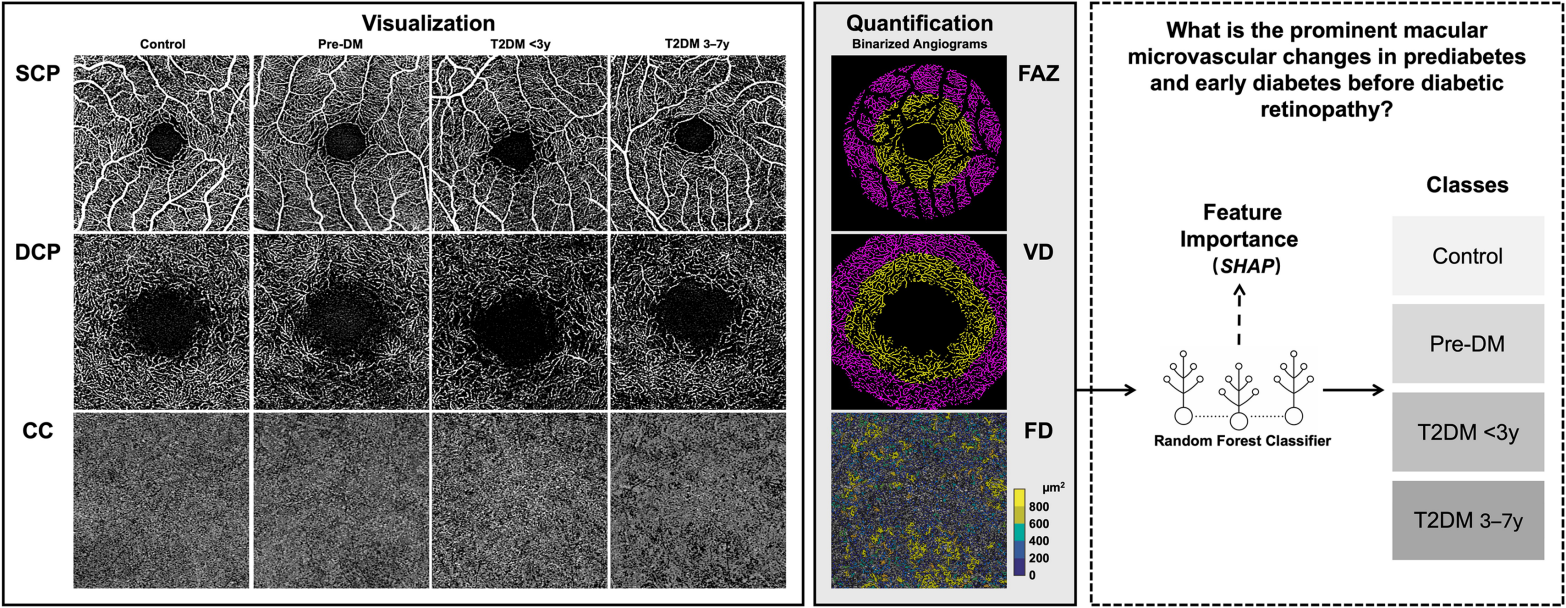
Study design, representative images, and analytical framework. SS-OCTA was used to visualize retinal and choroidal microvasculature, including the SCP, DCP, and choriocapillaris (CC). Representative en face SS-OCTA images of the SCP, DCP, and CC are shown for healthy controls, prediabetes (Pre-DM), type 2 diabetes mellitus duration <3 years (T2DM <3y), and T2DM duration 3–7 years (T2DM 3–7y). To ensure representative image selection, the eye with an overall CC FD percentage closest to the group median was selected from each group; the SCP, DCP, and CC images shown for each group were obtained from the same selected eye. Quantitative microvascular features were extracted from OCTA images and used as input variables for a random forest classifier to differentiate among healthy controls, Pre-DM, T2DM <3y, and T2DM 3–7y. Feature importance derived from the trained models was used to identify microvascular parameters contributing to classification across different stages of dysglycemia.

FAZ segmentation in SCP and DCP was performed using a U-Net-based deep learning model. FAZ area, perimeter, and acircularity were measured for the SCP only because of indistinct FAZ boundaries in the DCP. Two annular analysis zones (500 µm inner annulus and 1000 µm outer annulus) were generated around the FAZs in both SCP and DCP (Fig. 1).^11^ Images were binarized using a global threshold, from which PD was calculated as the proportion of perfused pixels per annulus area. Binarized maps were skeletonized to a 1-pixel width to derive VD, expressed as skeletonized vessel length per area.

Choriocapillaris images were compensated using structural slabs and binarized using a mean-SD threshold definition of FD.^12^ FD density was calculated for all sizes and for size-selective FD with thresholds of ≥200, ≥400, ≥600, and ≥800 µm^2^, respectively.

One eye per participant was included in the analysis. When both eyes were gradable, the right eye was preferentially used. Exclusion criteria included poor image quality because of low signal strength index, defocus, or motion artifacts; ocular conditions that may affect retinal microvasculature, including glaucoma, age-related macular degeneration, retinal vein occlusion, epiretinal membrane, or other retinal diseases; and axial length > 26.0 mm.

### Statistical Analysis

Retinal and choriocapillaris parameters were compared across four groups: (1) healthy controls; (2) prediabetes (pre-DM); (3) type 2 diabetes duration < 3 years (T2DM <3y); (4) type 2 diabetes duration 3–7 years (T2DM 3–7y). T2DM duration was defined as the interval between the date of diabetes diagnosis and SS-OCTA imaging.

Statistical analysis was conducted using R (version 4.4.1). Continuous variables were summarized as mean ± SD and categorical variables as *n* (%). Group comparisons were performed using ANOVA. Significant findings were further examined using post hoc pairwise comparisons. To correct for multiple testing and reduce false-positive error, *P*-values were adjusted using the Bonferroni method. Linear trend analysis treated ordinal DM duration groups as continuous independent variables, with adjustment for potential confounders, including age, sex, blood pressure, body mass index (BMI), and axial length. A two-sided *P* < 0.05 was considered statistically significant.

### Machine Learning and Feature Importance

To quantify the contribution of individual retinal microvasculature features to glycemic status classification, a random forest classifier was implemented. (Fig. 1) Three binary classification tasks were constructed to address microvascular involvement across glycemic stages: (1) pre-DM versus healthy controls; (2) T2DM <3y versus pre-DM; and (3) T2DM 3–7y versus pre-DM. Based on clinical rationale and study objectives, the final feature set included SCP VD, DCP VD, any-size FD of choriocapillaris, large-size FD (≥800 µm^2^) of choriocapillaris, and FAZ acircularity.

Model development and evaluation were conducted using stratified fivefold cross-validation to preserve class proportions across folds. For each outer fold, hyperparameters were tuned using grid search on the training portion only, and the optimized model was subsequently evaluated on the held-out test fold to avoid information leakage. Classification performance was summarized on the test folds using a default probability threshold of 0.5 for class assignment, and F1-score, precision, and recall.

Feature contributions were quantified using SHapley Additive exPlanations (SHAP). SHAP values were computed with TreeExplainer for the out-of-fold test samples in each cross-validation split. Out-of-fold SHAP values from all folds were then aggregated to provide overall feature importance and to visualize the direction and magnitude of each feature's association with the model output in SHAP summary plots.

## Results

### Patient Characteristics

A total of 309 eyes were analyzed, comprising 79 healthy controls, 85 individuals with pre-DM, 75 with T2DM <3y, and 70 with T2DM 3–7y. Seven eyes presented with minimal microaneurysms on color fundus images including three eyes in pre-DM, two eyes in with T2DM <3y, and two eyes with T2DM 3–7y.

No significant differences were observed in age, sex, or blood pressure across the groups. However, the distribution of ethnicity differed significantly among the groups (*P* = 0.008), with a higher proportion of Chinese participants in the pre-DM group and a lower proportion in the T2DM 3–7 years group. Notably, patients with prediabetes and DM had higher BMI values (*P* < 0.001). Importantly, axial length did not differ significantly among the groups (Table 1).

**Table 1.** Characteristics of Study Participants

Characteristic	Control (*n* = 79)	Pre-DM (*n* = 85)	T2DM <3 Y (*n* = 75)	T2DM 3–7 Y (*n* = 70)	*P* Value
Age (years)	59.3 ± 9.0	59.9 ± 7.3	59.8 ± 7.2	59.5 ± 8.9	0.956
Gender					
Female	46 (58.2%)	39 (45.9%)	37 (49.3%)	27 ± 38.6	0.112
Male	33 (41.8%)	46 (54.1%)	38 (50.7%)	43 ± 61.4	
Ethnicity					0.008
Chinese	65 (82.3%)	76 (89.4%)	61 (81.3%)	51 (72.9%)	
Indian	7 (8.9%)	3 (3.5%)	11 (14.7%)	9 (12.9%)	
Malay	2 (2.5%)	6 (7.1%)	1 (1.3%)	5 (7.1%)	
Others	5 (6.3%)	—	2 (2.7%)	5 (7.1%)	
BMI	23.7 ± 3.3	27.5 ± 4.2	28.4 ± 4.2	29.0 ± 4.7	<0.001
HbA1c^*^	NA	5.9% ± 0.4%	6.7% ± 0.5%	6.9% ± 0.7%	<0.001
Systolic BP (mm Hg)	135.2 ± 17.9	133.9 ± 16.0	134.7 ± 18.2	135.6 ± 16.6	0.942
Diastolic BP (mm Hg)	75.0 ± 10.6	75.8 ± 9.3	74.5 ± 10.1	74.1 ± 9.5	0.744
Axial Length (mm)	23.9 ± 1.0	24.0 ± 1.0	24.0 ± 1.1	24.2 ± 0.9	0.497

BP, blood pressure.

* HbA1c measurements were available only for participants with dysglycemia enrolled in the PREDICT EYE study. The *P* value was calculated among the three dysglycemia groups.

### Retinal Microvasculature Alterations

There were significant overall trends of decreasing VD and PD in both the SCP and DCP with worsening glycemic status and longer diabetes duration. Compared with healthy controls, individuals with prediabetes already demonstrated significantly reduced VD and PD in both plexuses, consistent across the inner and outer annulus. Although mean values further declined from prediabetes to T2DM <3y and from T2DM <3y to T2DM 3–7y, these differences did not reach statistical significance in either the SCP or DCP, regardless of annulus size (all *P* > 0.05). (Table 2; Supplementary Figure).

**Table 2.** Retinal Vvascular, FAZ, and Choriocapillaris Metrics Across Study Groups

					*P* Value					
Microvascular Parameters	Control (*n* = 79)	Pre-DM (*n* = 85)	T2DM <3 Y (*n* = 75)	T2DM 3–7 Y (*n* = 70)	Overall	Control vs. Pre-DM	Pre-DM vs. T2DM <3y	Pre-DM vs. T2DM 3–7y	T2DM <3y vs. T2DM 3–7y	*P* Value
VD of SCP inner Annulus	16.0% ± 1.1%	15.1% ± 1.4%	14.9% ± 1.5%	14.6% ± 1.5%	<0.001	<0.001	1	0.172	0.980	<0.001
VD of SCP outer Annulus	16.3% ± 1.3%	15.6% ± 1.6%	15.4% ± 1.5%	14.7% ± 1.8%	<0.001	0.005	1	0.007	0.096	<0.001
PD of SCP inner Annulus	29.3% ± 1.8%	28.4% ± 2.3%	28.0% ± 2.3%	27.6% ± 2.5%	<0.001	0.022	1	0.160	1	<0.001
PD of SCP outer Annulus	29.5% ± 2.2%	28.6% ± 3.1%	28.0% ± 2.6%	27.0% ± 3.0%	<0.001	0.127	0.770	0.010	0.184	<0.001
VD of DCP inner Annulus	16.1% ± 1.3%	15.5% ± 1.4%	15.3% ± 1.8%	15.1% ± 1.5%	<0.001	0.045	1	0.216	1	<0.001
VD of DCP outer Annulus	20.4% ± 1.4%	19.2% ± 1.7%	18.9% ± 2.1%	18.4% ± 1.7%	<0.001	<0.001	1	0.014	0.416	<0.001
PD of DCP inner Annulus	33.1% ± 2.7%	32.0% ± 3.1%	31.3% ± 3.7%	31.1% ± 3.6%	<0.001	0.084	0.586	0.322	1	<0.001
PD of DCP outer Annulus	41.4% ± 2.8%	39.0% ± 3.7%	38.1% ± 4.0%	37.1% ± 3.9%	<0.001	<0.001	0.607	0.012	0.556	<0.001
FAZ area (mm^2^)	0.3 ± 0.1	0.4 ± 0.1	0.3 ± 0.1	0.3 ± 0.1	0.021	0.16	0.044	0.038	1	0.556
FAZ perimeter (mm)	2.3 ± 0.3	2.4 ± 0.3	2.2 ± 0.4	2.2 ± 0.4	0.008	0.062	0.019	0.018	1	0.688
FAZ acircularity	1.1 ± 0.1	1.1 ± 0.1	1.1 ± 0.1	1.1 ± 0.1	0.704	1	1	1	1	0.573
CC FD density (any-size)	16.9% ± 0.8%	17.5% ± 0.8%	17.8% ± 1.1%	17.9% ± 1.0%	<0.001	<0.001	0.112	0.011	1	<0.001
CC FD density (large-size)	8.5% ± 0.9%	9.3% ± 0.9%	9.7% ± 1.1%	9.8% ± 1.0%	<0.001	<0.001	0.021	0.010	1	<0.001

CC, choriocapillaris.

Large-size FD, flow deficits with an area >800 µm^2^.

Notably, when participants with longer-duration diabetes (T2DM 3–7y) were compared with those with prediabetes, significant reductions in both VD and PD emerged, but only within the outer annulus. These included lower SCP VD (15.6 ± 1.6 vs. 14.7 ± 1.8, *P* < 0.01) and PD (28.6 ± 3.1 vs. 27.0 ± 3.0, *P* = 0.01), as well as lower DCP VD (19.2 ± 1.7 vs. 18.4 ± 1.7, *P* = 0.014) and PD (39.0 ± 3.7 vs. 37.1 ± 3.9, *P* = 0.012).

### FAZ Alterations

Differences were detected in FAZ area and perimeter across groups, mainly driven by the larger values observed in individuals with prediabetes compared with the other groups. In contrast, FAZ acircularity did not significantly differ among all groups (Table 2; Supplementary Figure).

### Choriocapillaris Alterations

There was a significant overall trend of increasing choriocapillaris FD density with worsening glycemic status and longer diabetes duration. Compared with healthy controls, individuals with prediabetes already exhibited increased FD density (*P* < 0.01). In contrast to the retinal vascular changes, choriocapillaris FD continued to significantly deteriorate beyond prediabetes, with a notable increase observed from prediabetes to recent-onset T2DM (any-size FD, *P* = 0.112; large-size FD, *P* = 0.02), and a further increase from prediabetes to longer-duration T2DM (any-size FD, *P* = 0.011; large-size FD, *P* = 0.01). However, no significant differences were found between recent-onset and longer-duration T2DM, regardless of FD size (all *P* > 0.05) (Table 2; Supplementary Figure).

### Machine Learning and Feature Importance

**Figure 2.**
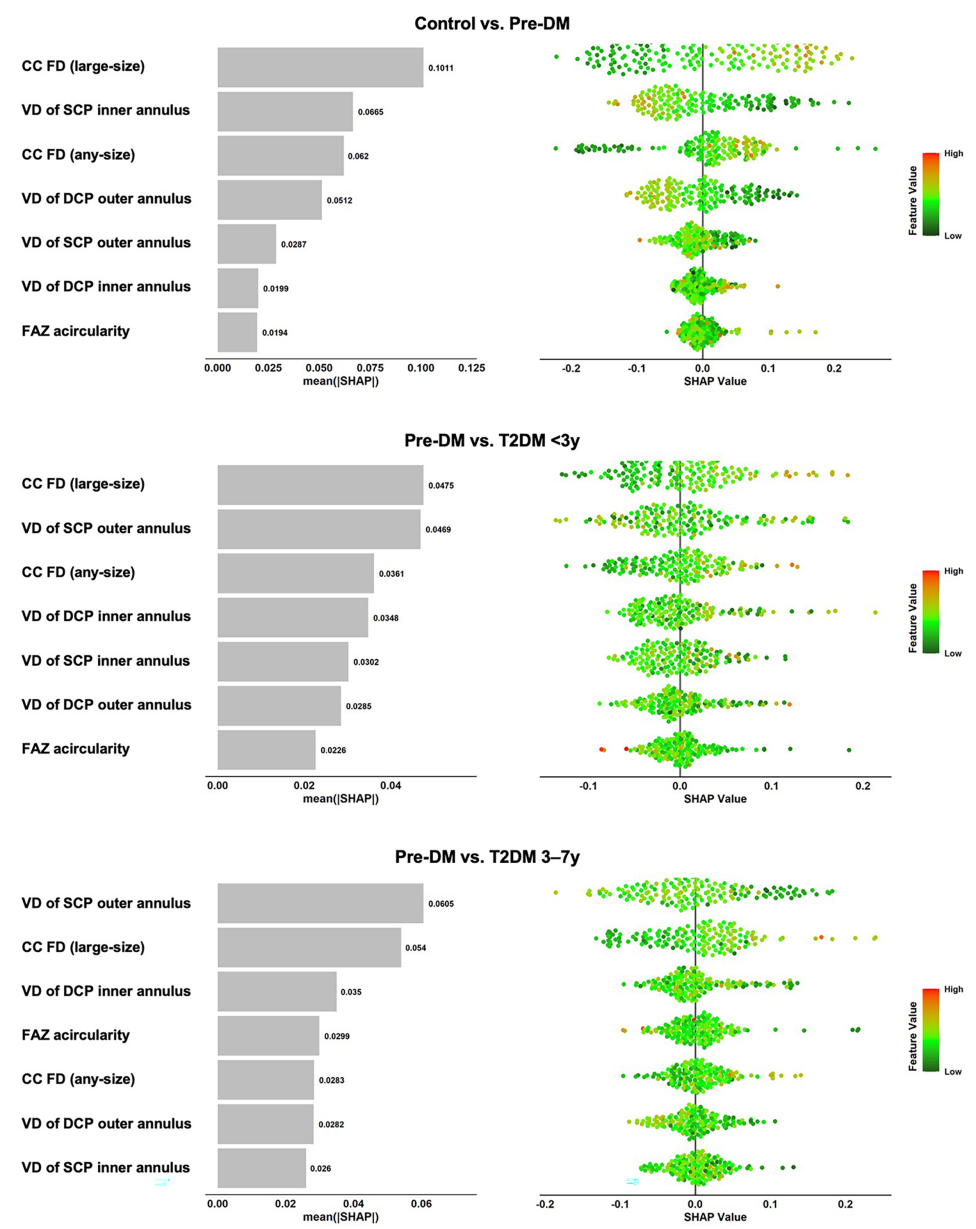
SHAP summary plots of feature importance. SHAP plots for the three classification tasks: prediabetes (Pre-DM) versus controls, T2DM duration <3 years (T2DM <3y) versus Pre-DM, and T2DM duration 3–7 years (T2DM 3–7y) versus Pre-DM. Features are ranked by mean absolute SHAP values (*left panels*), indicating their relative importance in the model. The right panels show the distribution of SHAP values for individual samples; each point represents one sample, with color indicating the corresponding feature value (*green*, low; *red*, high). Positive SHAP values indicate increased contribution toward the target class prediction, whereas negative values indicate the opposite.

The models showed moderate discrimination across the three classification tasks, as summarized in Table 3 (F1-score, precision, and recall). The SHAP results suggested that the relative contributions of OCTA features varied by glycemic stage (Fig. 2). Large-size FD ranked as the top contributor for distinguishing healthy controls from prediabetes and for separating recent-onset DM from prediabetes. Any-size FD was generally less important than large-size FD, but remained more influential than DCP VD and FAZ acircularity in the healthy controls versus pre-DM comparison. SCP VD was consistently among the highest-ranked features across tasks, whereas DCP VD became more prominent in comparisons involving longer-duration T2DM. FAZ acircularity was consistently ranked low across all classification tasks.

**Table 3.** F1 Score, Precision, and Recall for Glycemic Status Classification

Classification Task	F1 Score	Precision	Recall
Pre-DM vs. Control	0.68 ± 0.05	0.65 ± 0.08	0.72 ± 0.03
T2DM <3y vs. Pre-DM	0.54 ± 0.08	0.52 ± 0.06	0.58 ± 0.13
T2DM 3–7y vs. Pre-DM	0.59 ± 0.04	0.55 ± 0.03	0.65 ± 0.08

## Discussion

Our study demonstrates that microvascular alterations along the dysglycemia spectrum affect not only the retinal capillary plexuses but also, more prominently, the choriocapillaris. Multivariable adjustment confirmed that dysglycemia drives this choriocapillaris ischemia independently of the rising BMI in the prediabetic and diabetic cohorts. During the transition from prediabetes to early-onset diabetes, enlargement of large-size choriocapillaris FD emerged as a stand-alone distinguishing feature. With longer diabetes duration, significant reductions in retinal capillary PD and VD appeared in both SCP and DCP, particularly in the outer annulus, accompanied by a continued rise in choriocapillaris FD density.

To the best of our knowledge, this is the first study to report choriocapillaris alterations in individuals with prediabetes. Previous work by Kaya et al. demonstrated that increased choriocapillaris FD can be detected as early as the first year after diabetes diagnosis.^18^ Similarly, choriocapillaris perfusion in the macula has been shown to be reduced in patients with diabetes without DR compared with age-matched healthy controls, suggesting that impaired choriocapillaris perfusion may represent an early manifestation of subclinical diabetic vasculopathy.^22^ Our findings extend these observations by shifting the window of detection to an even earlier stage. In our cohort, choriocapillaris FD density was significantly increased in prediabetes regardless of FD size and further increased with increasing diabetes duration, particularly for large-size FD. The biological relevance of large-size FD has been proposed to reflect progressive choriocapillaris impairment, whereby smaller flow deficits coalesce into larger non-perfused areas, resulting in fewer but larger FD regions.^23^ This size-selective FD analysis may therefore provide greater sensitivity for detecting early microvascular impairment.

The choriocapillaris may be particularly vulnerable to metabolic stress induced by dysglycemia. As the terminal vascular layer of the ocular microcirculation, it exhibits a segmental lobular architecture with limited anastomotic connections between adjacent lobules, making it especially susceptible to ischemic injury.^24^^,^^25^ In addition, the choriocapillaris is characterized by uniquely large fenestrations and sustains one of the highest blood flow rates per unit tissue in the body to meet the substantial metabolic demands of RPE and photoreceptors.^26^ These structural and physiological characteristics may partially explain why choriocapillaris impairment becomes a key feature in the early diabetes continuum. Increasing evidence also implicates inflammatory activation and structural remodeling processes in early choroidal microvascular alterations in diabetes. In a spontaneous T2DM animal model (Goto-Kakizaki rats), reduced choriocapillaris vascular density was accompanied by increased microglial activation in the outer retina and enhanced VEGFR2 immunoreactivity, suggesting concurrent inflammatory and angiogenic signaling changes within the retinal-choroidal complex.^27^ Complementary structural evidence has been reported in spontaneously diabetic cynomolgus macaques, in which optical coherence tomography demonstrated increased choriocapillaris thickness compared with age-matched controls, with similar thickening also observed in postmortem human eyes with non-proliferative DR.^28^ These findings are thought to reflect diabetes-related choroidal remodeling. Although thickness measurements do not directly capture microvascular perfusion, these experimental observations collectively support the concept that inflammatory activation and structural remodeling of the choriocapillaris may occur early in diabetes, potentially contributing to the perfusion impairment detected by OCTA before clinically detectable DR develops. In line with these biological insights, our machine-learning analysis further highlighted choriocapillaris alterations, particularly large-size FD, as the dominant features distinguishing prediabetic eyes from healthy controls. Taken together, these findings suggest that choriocapillaris dysfunction detectable by SS-OCTA may represent an early imaging biomarker of diabetes-related microvascular injury, preceding more overt alterations in the retinal capillary plexuses.

Consistent with previous studies, we also observed reduced retinal vascular density in individuals with prediabetes compared with non-diabetic controls.^4^^–^^8^^,^^29^ However, discrepancies remain regarding whether these alterations in prediabetes are confined to the SCP or involve both SCP and DCP. Using 4.5 × 4.5 mm^2^ SS-OCTA scans, Wang et al.^5^ reported a significant reducted VD only in SCP. In contrast, other studies using different scan patterns reported significantly lower VD in both SCP and DCP.^7^^,^^29^ The heterogeneity among studies may be attributable to differences in devices, scan patterns, segmentation algorithms, and sample sizes. In our cohort, prediabetes was associated with significant VD reductions in both SCP and DCP, with values comparable to early diabetes (T2DM <3y). Further reductions in VD and PD were observed in individuals with longer diabetes duration, supporting the concept that retinal perfusion progressively declines across the early stages of diabetes.

With regard to changes in the FAZ area, previous studies have generally reported no significant differences among healthy controls, individuals with prediabetes, and patients with diabetes without DR.^4^^,^^5^^,^^7^ In contrast, our study observed significantly larger FAZ area and perimeter in the prediabetes group. However, OCTA studies have demonstrated substantial physiological variability in FAZ size within normal populations, which may limit the sensitivity of these metrics for detecting early microvascular alterations.^30^^–^^32^ Pathological enlargement of the FAZ area and perimeter is more typically associated with the presence of DR, particularly in advanced stages such as proliferative DR.^16^^,^^25^ Consistent with this notion, FAZ acircularity, which has been suggested to be a more robust indicator because of its lower physiological variability, did not differ significantly across groups in our study.

Taken together with prior studies using color fundus photography and handheld electroretinography that have demonstrated early functional and structural abnormalities before clinically visible DR,^29^^,^^33^^,^^34^ our findings provide converging evidence that choriocapillaris and retinal microvascular injury may occur before the onset of clinically detectable DR. Importantly, current glycemic thresholds for diagnosing diabetes were originally established based on the appearance of overt DR. Increasing evidences now suggest that diabetes-specific end-organ complications may begin to develop even before these diagnostic criteria are met. Our findings raise the possibility that microvascular damage may already be present during the prediabetic stage, suggesting that current glycemic thresholds may not fully capture the earliest phases of diabetes-related tissue injury. Future longitudinal studies in larger populations are warranted to determine whether alternative glycemic thresholds or imaging biomarkers may better reflect the onset of early microvascular damage.

A major strength of the present study is its well-charactered and systemic study design including the availability of precise diabetes duration, particularly during the early stages of the disease, which is rarely captured with accuracy in most previously published studies. Another key strength is the use of SS-OCTA, which allows reliable quantification of retinal microvasculature and, importantly, enables visualization and quantitative assessment of the choriocapillaris. Nevertheless, OCTA has inherent limitations in detecting and quantifying blood flow. OCTA resolves only a limited range of flow velocities and are not able to reliably distinguish different flow speeds within its detectable range. Consequently, OCTA-derived vascular metrics may not fully reflect subtle reductions in capillary perfusion in the absence of frank capillary dropout. Therefore the apparent plateau in retinal capillary alterations observed in this study should be interpreted as a plateau in the OCTA-derived metrics rather than definitive evidence that retinal microvascular dysfunction ceases to progress. However, the generalizability of our findings to other populations remains uncertain, because the study cohort primarily comprised individuals of Chinese, Malay, and Indian ethnicity, representing a Southeast Asian population.

## Conclusions

Macular microvascular impairment involving both the retinal capillary plexuses and the choriocapillaris is detectable as early as the prediabetic stage. Feature-importance analysis effectively identified stage-specific patterns of macular microvascular involvement across different dysglycemic states and diabetes durations. Collectively, these findings suggest that early microvascular alterations may predominantly affect the choriocapillaris, particularly large-size FD, before progressive involvement of the retinal capillary plexuses becomes evident with longer diabetes duration.

## Supplementary Material

Supplement 1
