# Supplementary material for: Macular Choriocapillaris and Retinal Microvascular Alterations Across the Prediabetes–Early Type 2 Diabetes Continuum
Source: Invest Ophthalmol Vis Sci. 2026 Aug 6;67(10):16. doi: 10.1167/iovs.67.10.16 (PMC13452433; doi:10.1167/iovs.67.10.16)
Supplement: Supplementary file 1 [file iovs-67-10-16_s001.docx]

**Supplementary Figure:** Box plots of macular OCTA metrics across healthy controls, prediabetes (Pre-DM), type 2 diabetes mellitus duration <3 years (T2DM <3y), and T2DM duration 3–7 years (T2DM 3–7y).

(A-D) Superficial capillary plexus (SCP) metrics. VD and PD in the inner annulus (A,B) showed significant reductions between controls and Pre-DM. VD and PD in the outer annulus (C,D) showed significant reductions between controls and Pre-DM and between Pre-DM and T2DM 3–7y.

(E-H) Deep capillary plexus (DCP) metrics. VD in the inner annulus (E) showed a significant reduction between controls and Pre-DM, whereas PD (F) showed no significant differences. In the outer annulus, both VD and PD (G,H) showed significant reductions between controls and Pre-DM and between Pre-DM and T2DM 3–7y.

(I-J) Foveal avascular zone (FAZ) metrics. FAZ area (I) was larger in Pre-DM without a clear trend across groups, while FAZ acircularity (J) showed no significant differences.

(K-L) Choriocapillaris (CC) flow deficit (FD) metrics. Any-size FD density (K) increased significantly between controls and Pre-DM and between Pre-DM and longer-duration T2DM. Large-size FD density (L) showed a progressive increase from controls to Pre-DM, early T2DM, and longer-duration T2DM.
